# Supplementary material for: Diagnosing the performance of food systems to increase accountability toward healthy diets and environmental sustainability
Source: PLoS One. 2022 Jul 29;17(7):e0270712. doi: 10.1371/journal.pone.0270712 (PMC9337654; doi:10.1371/journal.pone.0270712)
Supplement: S1 Table — (DOCX) [file pone.0270712.s001.docx]

**S1 Table. Diagnosis indicators where thresholds were established using public health guidance (Cutoff Type 1).**

| **Sector** | **Subsector** | **Indicator** | **Source** | **Public health guidance or**  **recommendations** |
| --- | --- | --- | --- | --- |
| Food Security, Diets and Nutrition | Nutritional status | 23. Prevalence of under-5 stunting (HAZ <-2 SD) (%) | UNICEF, WHO, and World Bank (40) | Prevalence thresholds for stunting in children under 5 have been proposed by De Onis et al. (50). Less than 10% prevalence of stunting was determined to be of low public health significance while greater than 20% was determined to be of high public health significance. |
|  |  | 24. Prevalence of under- 5 wasting (WHZ < -2 SD) (%) | UNICEF, WHO, and World Bank (40) | Prevalence thresholds for wasting in children under 5 have been proposed by De Onis et al.(50). Less than 5% prevalence of wasting was determined to be of low public health significance while greater than 10% was determined to be of high public health significance. |
|  |  | 25. Prevalence of underweight in women (BMI <18.5 kg/m^2^) (%) | NCD RisC (41) | Prevalence thresholds for thinness in women of reproductive age have been proposed by the WHO^1^. A prevalence of 5-10% of thinness was considered a warning sign, while greater than 10% was determined to be a poor situation. |
|  |  | 26. Prevalence of anemia in women 15-49 years (%) | WHO Global Health Observatory (42) | Prevalence thresholds for anemia in women of reproductive age have been proposed by the WHO (2008)^2^. Less than 20% prevalence of anemia was determined to be of mild public health concern while greater than 40% was determined to be a high public health concern. |
|  |  | 27. Prevalence of under-5 overweight and obesity (WHZ >2 SD) (%) | UNICEF, WHO, and World Bank (40) | Prevalence thresholds for overweight and obesity in children under 5 have been proposed by De Onis et al. (50). Less than 5% prevalence of overweight and obesity was determined to be of low public health significance while greater than 10% was determined to be of high public health significance. |

**^1^** WHO Expert Committee. Physical status: the use and interpretation of anthropometry: report of a WHO Expert Committee. Geneva: World Health Organization; 1995.

^2^ WHO. Worldwide prevalence of anaemia 1993-2005 of: WHO Global Database on Anaemia [Internet]. de Benoist B, McLean E, Egli I, Cogswell M, editors. Geneva: World Health Organization; 2008 [cited 2020 Feb 17]. Available from: http://whqlibdoc.who.int/publications/2008/9789241596657_eng.pdf
