# Supplementary material for: Diagnosing the performance of food systems to increase accountability toward healthy diets and environmental sustainability
Source: PLoS One. 2022 Jul 29;17(7):e0270712. doi: 10.1371/journal.pone.0270712 (PMC9337654; doi:10.1371/journal.pone.0270712)
Supplement: S2 Table — (DOCX) [file pone.0270712.s002.docx]

**S2 Table.** **Diagnosis indicators where thresholds were established using public health and environmental recommendations (Cutoff Type 2).**

| **Sector** | **Subsector** | **Indicator** | **Source** | **Public health guidance or**  **recommendations** |
| --- | --- | --- | --- | --- |
| Food supply chains | Production systems and input supply | 1. Crop species richness (average number of crops/ unit of land) | IFPRI 2019 (34) | According to Sirami et al.^1^, agricultural landscapes with larger crop diversity (min 0- max 9) host larger multi-taxonomic diversity.  Countries with an average crop diversity of seven or fewer signifies a likely and potential challenge area for the country’s food system. |
| Food environment | Food availability | 6. Dietary energy in the food supply (kcal/capita/d) | FAOSTAT (35) | To achieve energy intake recommendations established by the WHO^2^ and EAT-Lancet (6) reference diets, dietary energy in the food supply needs to be greater than 2500 kcal/capita/day. |
|  |  | 8. Fruit supply (g/capita/d) | FAOSTAT (35) | To achieve fruit and vegetable intake recommendations established by the WHO (65) and EAT-Lancet (6) reference diets, fruit supply needs to be greater than 200 g/capita/day. |
|  |  | 9. Vegetable supply (g/capita/d) | FAOSTAT (35) | To achieve fruit and vegetable intake recommendations established by the WHO (65) and EAT-Lancet (6) reference diets, vegetable supply needs to be greater than 200 g/capita/day. |
|  |  | 10. Pulse supply (g/capita/d) | FAOSTAT (35) | To achieve pulse optimal intake suggested by the Global Burden of Disease (15), supply of pulses needs to be greater than 60 g/capita/day. |
|  | Food affordability | 15. Cost of an energy sufficient diet (2011USD/capita/d) | Food Prices for Nutrition (38) | The food portion of the international poverty line was identified as $1.20 (38), and is currently determined based on the cost of adequate dietary energy. The cost of an energy sufficient diet should be no more than $1.20 in international dollars. |
|  |  | 13. Relative cost of adequate legumes, nuts, and seeds  (ratio of the cost of the recommended amount of legumes, nuts, and seeds to the cost of the recommended amount of starchy staples per person per day) | Food Prices for Nutrition (38) | Legumes have similar storage properties as grains, so the relative cost of legumes compared to grains should be similar, other supply side issues equal. |
|  |  | 13. Affordability of a healthy diet (ratio of the cost of a healthy diet to observed per capita food expenditures from national accounts) | Food Prices for Nutrition (38) | For people to access a healthy diet, the cost of a healthy diet should not be more than typical food expenditures. |
| Environment Outcomes | Consumption Level | 35. Total ecological footprint of consumption (global ha/ capita) | Global Footprint Network (46) | An ecological footprint equivalent to the world’s biocapacity in 2014 (1.67 gha per capita)^3^ indicates a globally sustainable demand of resources. Therefore, countries with an ecological footprint above 1.68, and in particular, above the global average of 2.75 gha in 2016, signifies a likely and potential challenge area for the country’s food system. |
|  | Measures at Production Level | 36. Total ecological footprint of production (global hectares/capita) | Global Footprint Network (46) | An ecological footprint equivalent to the world’s biocapacity in 2014 (1.67 gha per capita)^3^ indicates a globally sustainable demand for resources. Therefore, countries with an ecological footprint above 1.67, and in particular, above the global average of 2.75 gha in 2016, signifies a potential and likely challenge area for the country’s food system. |
|  |  | 37. Average number of threats to soil biodiversity | Orgiazzi et al. 2016 (47) | No global cutoffs currently exist in the literature for the average number of threats to soil biodiversity. The maximum average number of threats per country is around four, although this indicator accounts for eight threats.  Countries with an average number of threats above two signifies a likely challenge area for the country’s food system. |
|  |  | 38. Agricultural land change from 2008 to 2018 (log(1,000 ha/year)) | FAOSTAT (48) | No global cutoffs currently exist in the literature for agricultural land change. The absolute change was log-transformed to overcome the long tails of the normal distribution. Countries in these extreme ends signal a rapid abandon or expansion of agricultural land, hence potential linkages with land degradation or deforestation. Countries with “stable” agricultural land have ranged  >-1 & < 1 * 1000 hectares per year (log(abs(<=0). Countries with an extremely dynamic agricultural land expand or contract <-100 or >100* 1000 hectares per year (log(abs(>2). |
|  |  | 39. Average proportion of agricultural lands embedding at least 10% of natural vegetation (%) | Jones et al., 2021 (49) | According to DeClerck et al.^4^, all agricultural lands should embed at least 10% of natural vegetation to guarantee ecosystem functioning. Countries with less than 25% of their agricultural land not embedding 10% of natural vegetation signifies a likely challenge area for the country’s food system. |

^1^ Sirami C, Gross N, Baillod AB, Bertrand C, Carrié R, Hass A, et al. Increasing crop heterogeneity enhances multitrophic diversity across agricultural regions. Proc Natl Acad Sci. 2019 Aug 13;116(33):16442–7.

^2^ Schneider K and Herforth A. Software tools for practical application of human nutrient requirements in food-based social science research [version 1; peer review: 1 approved with reservations, 1 not approved]. *Gates Open Res* 2020, **4**:179 (<https://doi.org/10.12688/gatesopenres.13207.1>)

^3^ Atkinson G, Dietz S, Neumayer E, Agarwala M, editors. Handbook of sustainable development. Second edition. Cheltenham, UK: Edward Elgar; 2014. 590 p.

^4^ Fabrice DeClerck, Sarah Jones, Natalia Estrada-Carmona, Alexander Fremier. Spare half, share the rest: A revised planetary boundary for biodiversity intactness and integrity. Biological Sciences. Article pre-print 2021
