# Supplementary material for: Diagnosing the performance of food systems to increase accountability toward healthy diets and environmental sustainability
Source: PLoS One. 2022 Jul 29;17(7):e0270712. doi: 10.1371/journal.pone.0270712 (PMC9337654; doi:10.1371/journal.pone.0270712)
Supplement: S3 Table — (DOCX) [file pone.0270712.s003.docx]

**S3 Table. Diagnosis indicators where thresholds were established by consulting histogram of global distribution (Cutoff Types: 3 (unimodal) & 4 (bimodal or skewed)).**

| **Sector** | **Subsector** | **Indicator** | **Source** | **Histogram** |
| --- | --- | --- | --- | --- |
| Food supply chains | Storage and distribution | 2. Cereal losses (% of domestic supply) | FAOSTAT (35) | 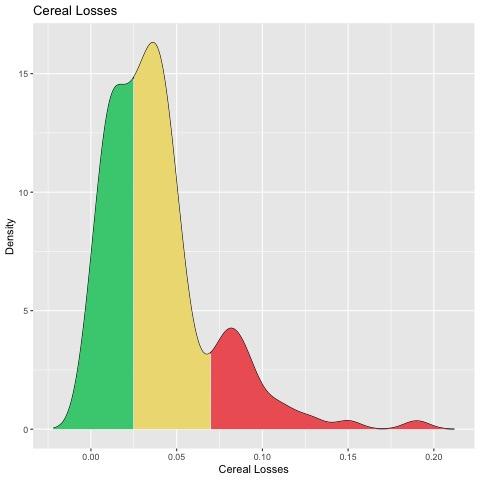 |
|  |  | 3. Pulse losses (% of domestic supply) | FAOSTAT (35) | 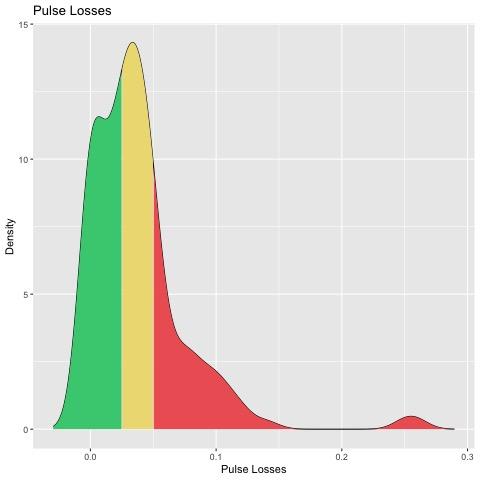 |
|  |  | 4. Fruit losses (% of domestic supply) | FAOSTAT (35) | 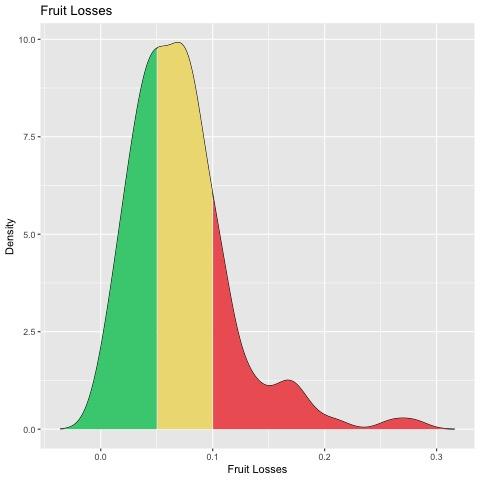 |
|  |  | 5. Vegetable losses (% of domestic supply) | FAOSTAT (35) | 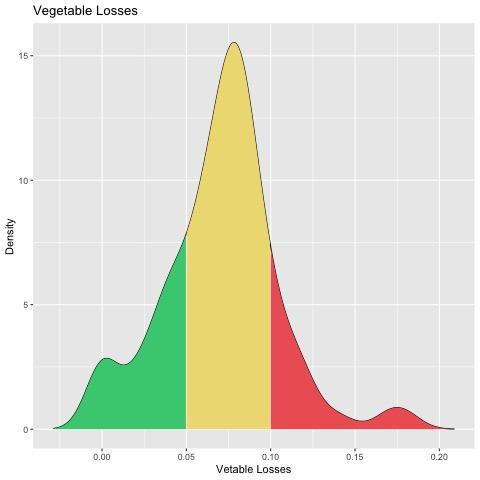 |
| Food environment | Food availability | 7. Dietary energy from cereals, roots, and tubers (%) | FAOSTAT (35) | 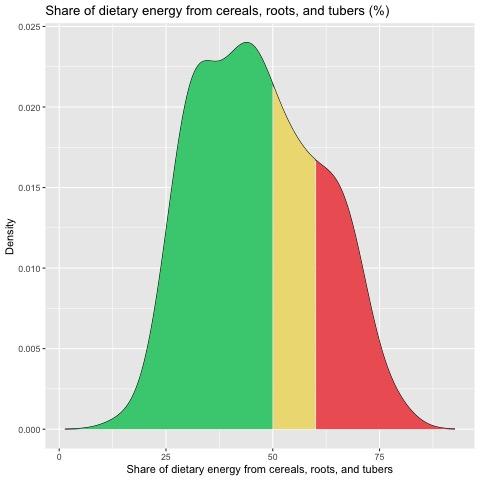 |
|  | Product properties | 11. Retail value of ultra-processed foods (USD/capita/year) | Euromonitor (37) | 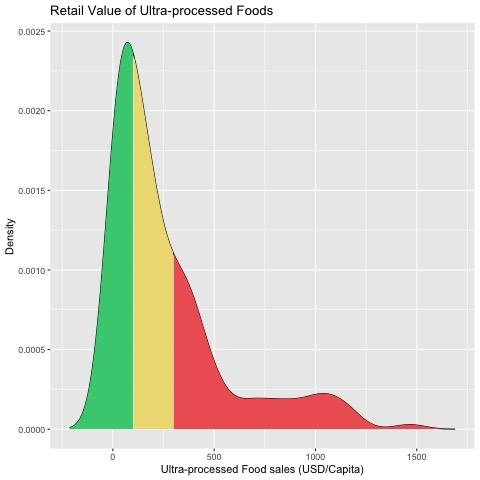 |
|  | Food Affordability | 12. Relative cost of adequate fruits and vegetables (ratio of the daily cost of adequate fruits and vegetables to the daily cost of adequate starchy staples) | Food Prices for Nutrition (38) | 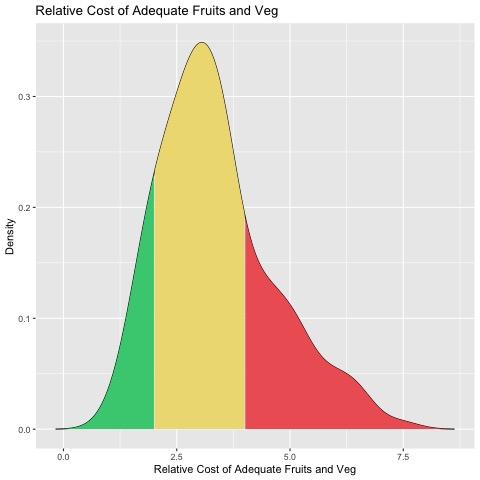 |
|  |  | 14. Relative cost of healthy diet ( ratio of the cost of a healthy diet to an energy sufficient diet) | Food Prices for Nutrition (38) | 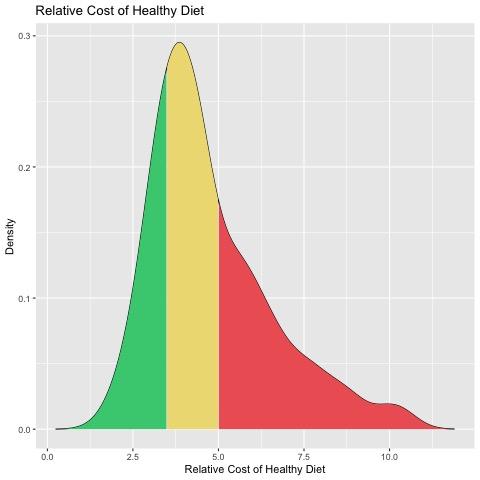 |
| Food Security, Diets and Nutrition | Food Security | 17. People who cannot afford a healthy diet (%) | Food Prices for Nutrition (38) | 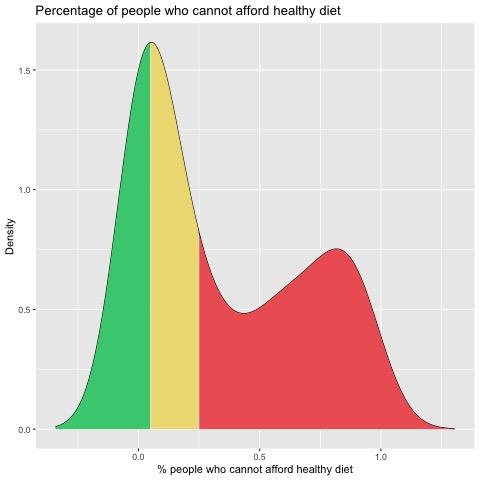 |
|  |  | 18. Prevalence of moderate or severe food insecurity (%)  (FIES) | FAOSTAT (36) | 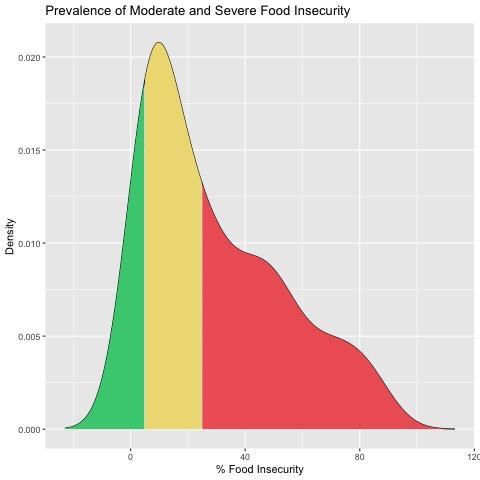 |
|  |  | 28. Prevalence of undernourishment (%) | FAOSTAT (36) | 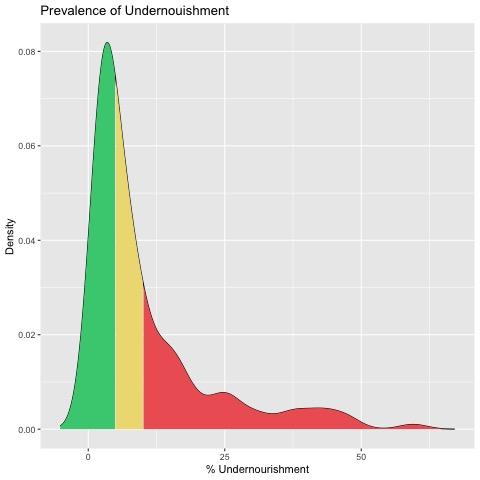 |
|  | Dietary intake | 20. Prevalence of minimum diet diversity (MDD) in infants age 6-23 months (%) | UNICEF (39) | 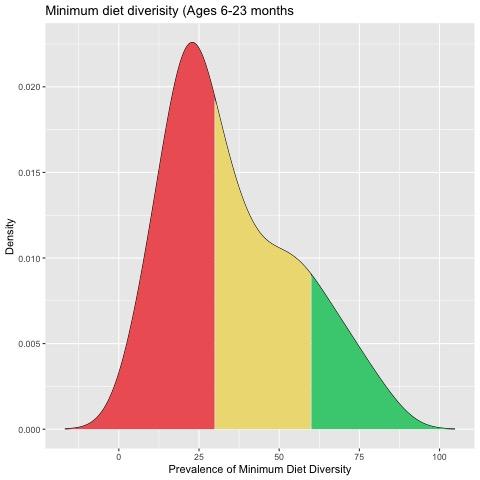 |
|  |  | 21. Prevalence of infants (6-23 months) consuming zero fruits and vegetables (%) | UNICEF (39) | 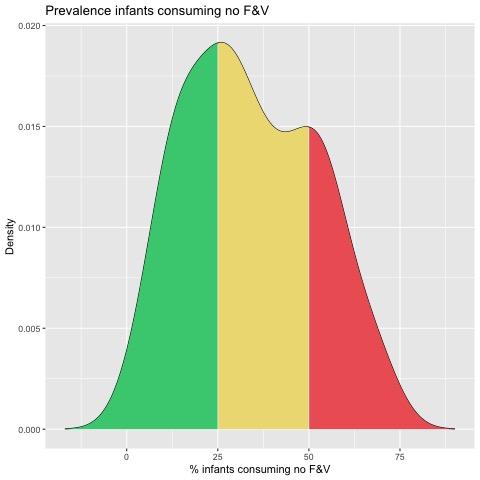 |
|  |  | 22. Prevalence of infants (6-23 months) consuming no flesh foods (%) | UNICEF (39) | 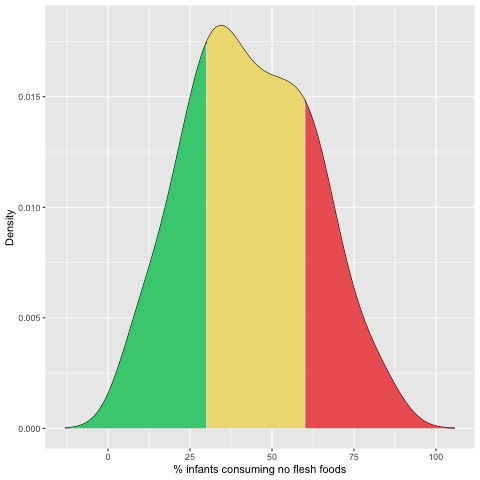 |
|  | Nutritional status | 28. Prevalence of adult obesity (BMI ≥ 30 kg/m^2^) (%) | NCD RisC (41) | 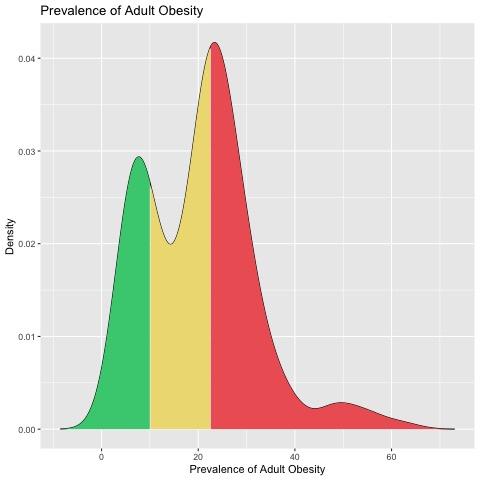 |
| Food Security, Diets and Nutrition | NCDs | 29. Prevalence of adult raised blood pressure (SBP ≥ 140 or DBP ≥ 90 mm Hg) (%) | NCD RisC (41) | 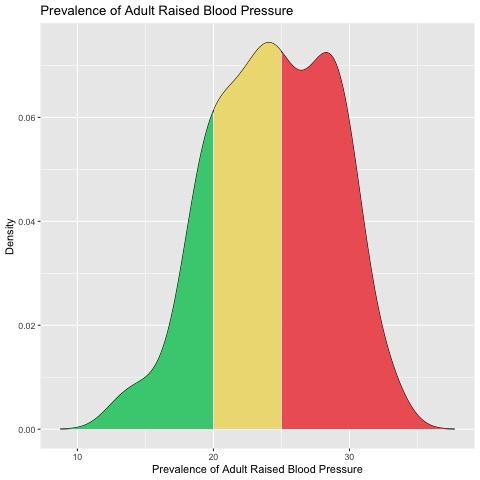 |
|  |  | 30. Prevalence of diabetes (%) | NCD RisC (41) | 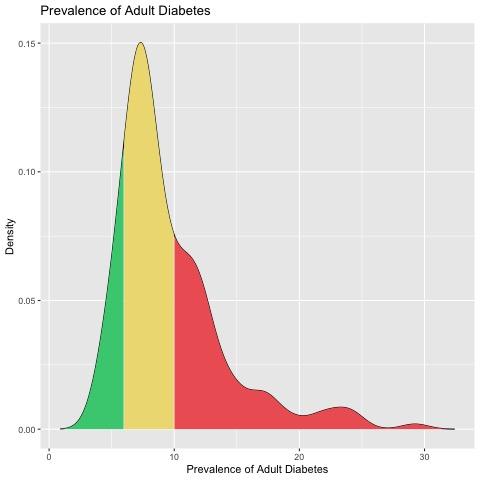 |
| Environment Outcomes | Environment Measures at Consumption Level | 31. GHG emissions of food consumption (kg CO_2_-equivalent / capita) | WWF (45) | 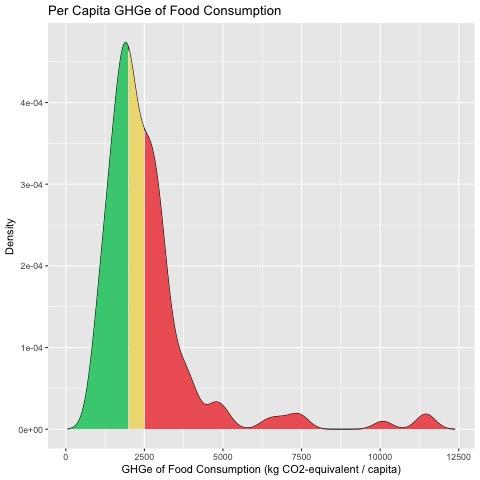 |
|  | Environment Measures at Production Level | 32. Water use linked to food consumption (liters/capita) | WWF (45) | 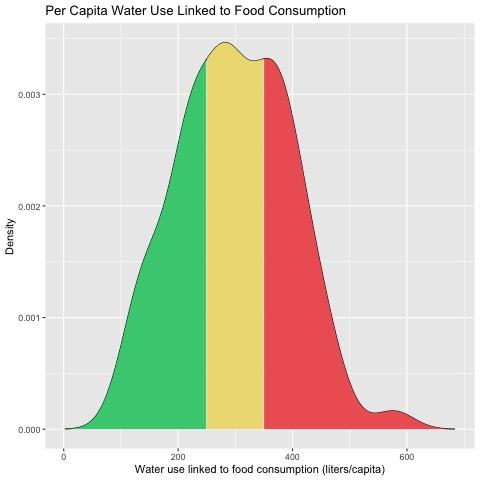 |
|  |  | 33. Eutrophication of food consumption (g PO_4_-equivalent /capita) | WWF (45) | 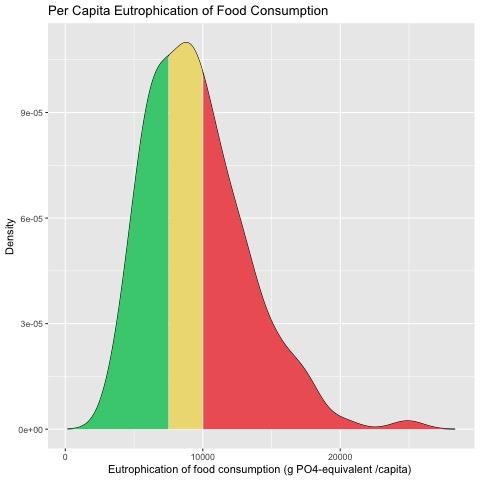 |
|  |  | 34. Biodiversity impact of food consumption (extinctions per species year*10^12^/capita) | WWF (45) | 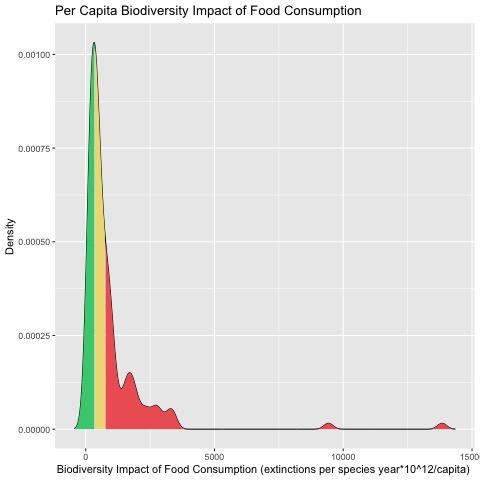 |
